# Interferon-γ Produced by Microglia and the Neuropeptide PACAP Have Opposite Effects on the Viability of Neural Progenitor Cells

**DOI:** 10.1371/journal.pone.0011091

**Published:** 2010-06-14

**Authors:** Johanna Mäkelä, Raili Koivuniemi, Laura Korhonen, Dan Lindholm

**Affiliations:** 1 Minerva Medical Research Institute, Biomedicum-2 Helsinki, Helsinki, Finland; 2 Institute of Biomedicine/Biochemistry, University of Helsinki, Helsinki, Finland; University of North Dakota, United States of America

## Abstract

Inflammation is part of many neurological disorders and immune reactions may influence neuronal progenitor cells (NPCs) contributing to the disease process. Our knowledge about the interplay between different cell types in brain inflammation are not fully understood. It is important to know the mechanisms and factors involved in order to enhance regeneration and brain repair. We show here that NPCs express receptors for interferon-γ (IFNγ), and IFNγ activates the signal transducer and activator of transcription (STAT) protein-1. IFNγ reduced cell proliferation in NPCs by upregulation of the cell cycle protein p21 as well as induced cell death of NPCs by activating caspase-3. Studies of putative factors for rescue showed that the neuropeptide, Pituitary adenylate cyclase-activating polypeptide (PACAP) increased cell viability, the levels of p-Bad and reduced caspase-3 activation in the NPCs. Medium from cultured microglia contained IFNγ and decreased the viability of NPCs, whilst blocking with anti-IFNγ antibodies counteracted this effect. The results show that NPCs are negatively influenced by IFNγ whereas PACAP is able to modulate its action. The interplay between IFNγ released from immune cells and PACAP is of importance in brain inflammation and may affect the regeneration and recruitment of NPCs in immune diseases. The observed effects of IFNγ on NPCs deserve to be taken into account in human anti-viral therapies particularly in children with higher rates of brain stem cell proliferation.

## Introduction

The nervous system interacts with the immune system during inflammation that is part of many neurodegenerative diseases. Cytokines secreted by immune cells mediate the effects of inflammation in the brain. Increased production of cytokines is observed in different brain disorders in experimental animals and in humans [1]. Our knowledge about the inflammatory process in the brain and the interplay between different cell types in inflammation are not fully understood [1]–[3]. It is important to know the different mechanisms and factors that underlie cell reactions in brain in order to enhance regeneration and brain repair.

NPCs are present in the developing neuroepithelium and in neurogenic areas in the adult brain [4], [5]. NPCs are self-renewing cells that give rise to neuroblast and glial cells in the nervous system. Different factors in the local milieu influence cell proliferation and differentiation of NPCs [6]–[8]. NPCs have been shown to react to tissue trauma as a part of the defense mechanism. Chronic inflammation was shown to impair neurogenesis and negatively influence neuronal stem cells in the rodent hippocampus [2], [9]. In line with this, reduced brain inflammation using anti-inflammatory drugs restores neurogenesis in rat hippocampus [2] and after brain ischemia [10]. On the other hand, glucocorticoid hormones, which are increased after stress and immune activation, reduce neurogenesis and the proliferation of NPCs [11]. The roles of different cytokines and their interactions in the regulation of NPCs are so far largely unknown.

In this work, we have studied the Interferons (IFN) family of cytokines, which are synthesized and secreted by different cells types during inflammation and in immune reactions [12]. We observed that NPCs express IFNγ receptors (IFNγR) *in vitro* and *in vivo*, and that stimulation with IFNγ activates STAT1 signaling in the NPCs. IFNγ caused a decrease in cell viability of NPCs accompanied by reduced cell proliferation and increased cell death. One source of IFNγ in the brain is microglial cells that produce increasing amounts of cytokines after cell activation [1], [3], [12], [13]. Cultivation of NPCs with medium from microglia decreased cell viability that was rescued by the addition of the neuropeptide PACAP. These results reveal an important interaction between NPCs and microglial cells that involves the cytokine IFNγ and neuropeptide PACAP and which is probably of importance in brain inflammation and disease.

## Results

### NPCs express receptors for IFNγ

**Figure 1:**
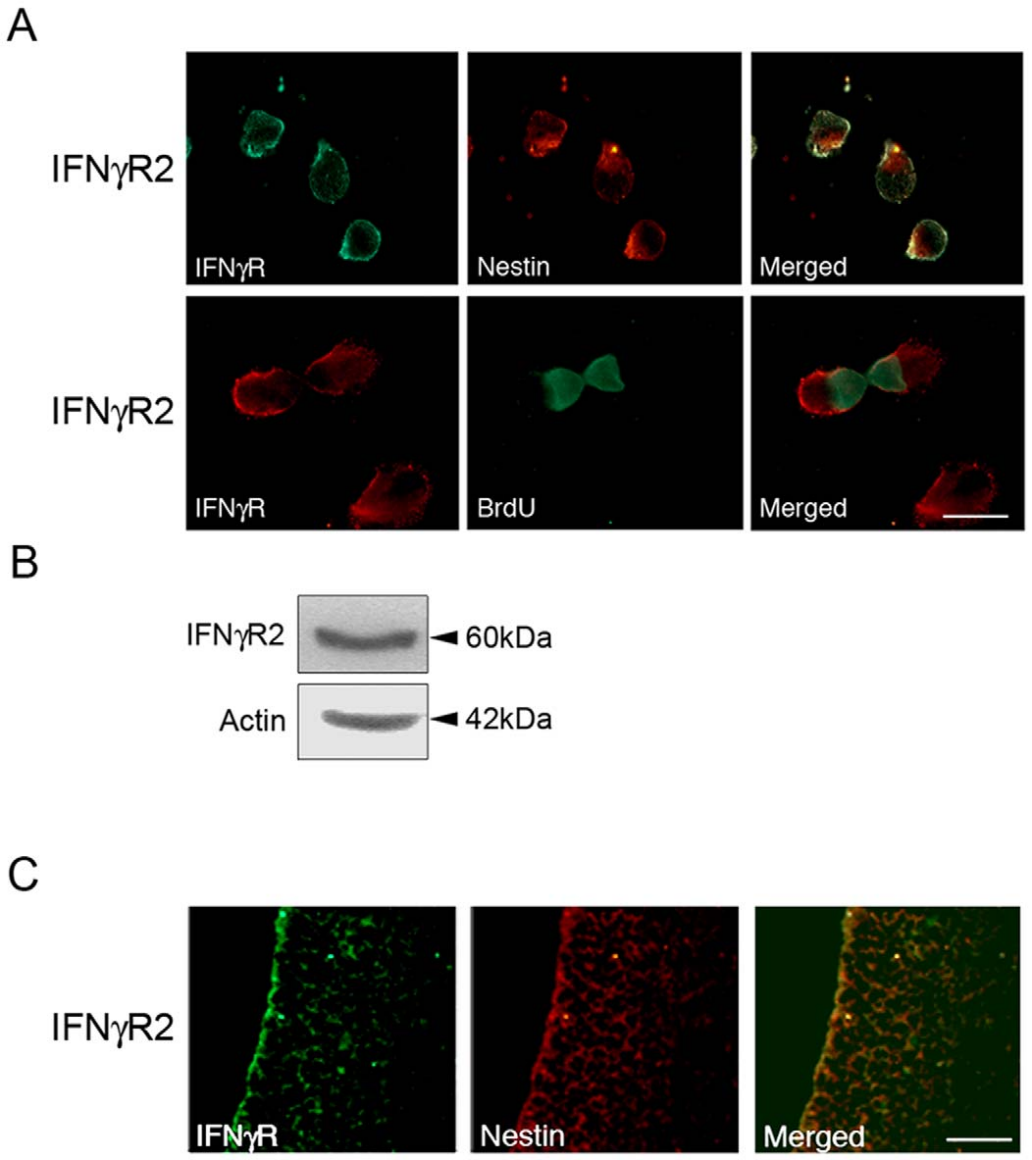
Neural progenitor cells express IFNγ receptors. NPCs were prepared from embryonic, E17 old rat brain and cultured as described in Methods. (**A**) Upper panel. Immunostaining using antibodies against the IFNγR2 receptor (green fluorescence) and against nestin (red fluorescence) as a marker for NPCs. Control without primary antibody showed no staining. Lower panel. BrdU labeling was done as described in Methods. Note expression of IFNγR2 in dividing NPCs. Scale bar, 10 µm. (**B**) Immunoblot shows the presence of IFNγR2. β-actin was used as control. (**C**) Sections from E17 rats were double-stained using antibodies against nestin and IFNγR2. Note coexpression in cells in neuroepithelium. Scale bar, 90 µm.

In the brain, astrocytes and neurons have been shown to express IFNγR [12], [14]. Immunostaining of embryonic NPCs cultured as neurospheres and colabelled with the marker nestin also expressed IFNγ receptor-2 (IFNγR2, Fig. 1A). Labeling with BrdU showed that the IFNγR2 positive cells actively divide in the cultures (Fig. 1a). Semiquantitative PCR confirmed the expression of the IFNγR-2 in the NPCs (Fig. 1B). IFNγR2 was also observed with immunoblotting of NPCs and the receptors were present in developing neuroepithelium containing nestin positive NPCs (Fig. 1C).

### IFNγ decreases viability of NPCs and affects cell proliferation

**Figure 2:**
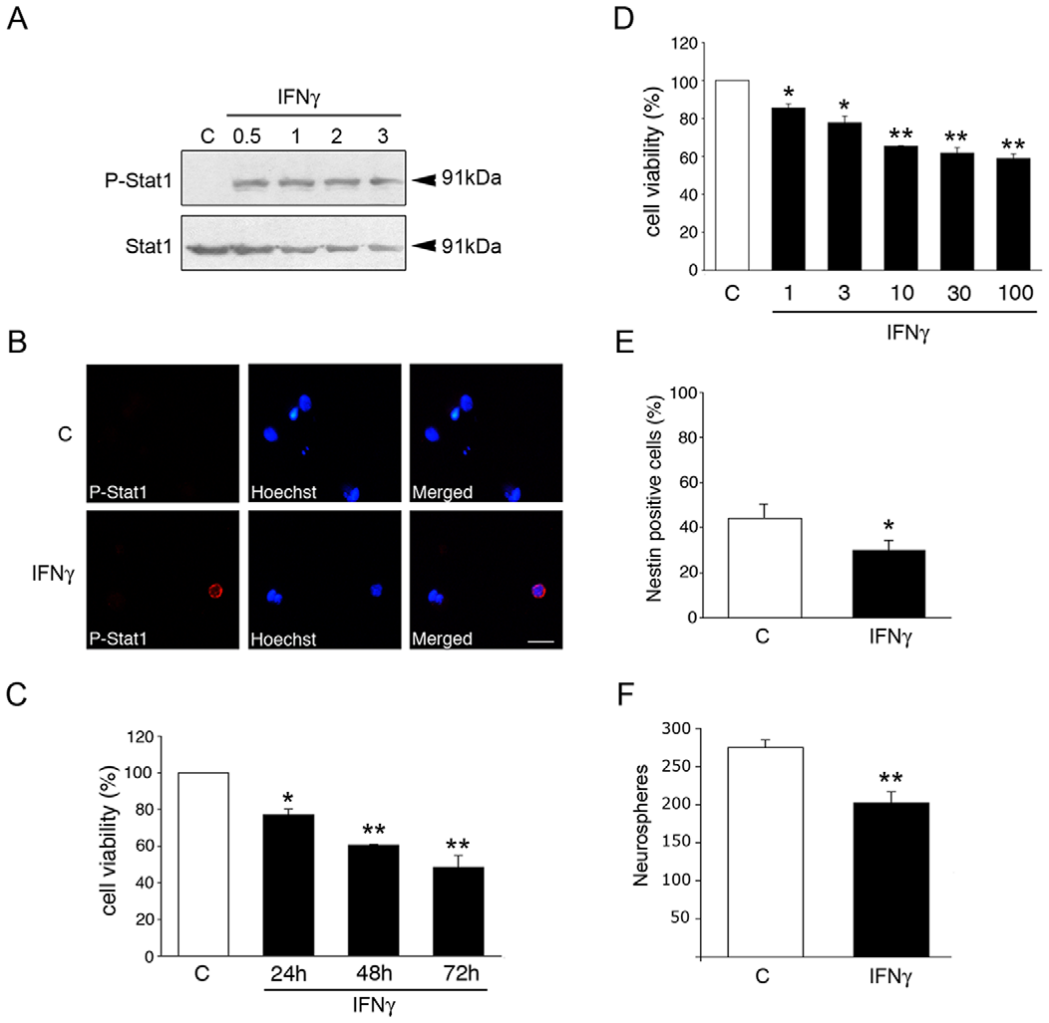
IFNγ activates STAT signaling and decreases cell viability of NPCs. NPCs were treated with 100 ng/ml IFNγ for different times and analyzed as described in Methods. *p<0.05 and **p<0.001 comparing IFNγ treated vs. controls. (**A**) Immunoblot shows increased levels of phospho-STAT1 (p-Stat1) after 0.5 h. (**B**) Immunostaining showed the presence of p-Stat1 in the nucleus after 2 h treatment. Scale bar, 10 µm. (**C**) Cell viability was determined by the MTT assay as described in Methods. Values are means ±SD, n = 4. (**D**) Dose response curve. Cells were treated for 48 h and cell viability determined. Values are means ±SD, n = 4. (**E**) Cells were treated for 48 h and the number of nestin positive NPCs was analyzed. Values are means ±SD, n = 4. (**F**) Neurospheres were dissociated and about 5000 cells per well were incubated for 3days in absence or presence of 100 ng/ml IFNγ. The number of secondary neurospheres formed was counted. Values are means ±SD, n = 4.

Treatment of NPCs with 100 ng/ml IFNγ induced the rapid phosphorylation of STAT1 (Fig. 2A), with translocation of the protein into the nucleus (Fig. 2B). IFNγ also decreased the viability of NPCs (Fig. 2C). Dose response curve showed that the half-maximum effect of IFNγ was about 3 ng/ml (Fig. 2D). Immunostaining confirmed that the number of nestin positive NPCs decreased after IFNγ treatment (Fig. 2E), and IFNγ reduced the number of secondary neurospheres formed in the cultures (Fig. 2F).

**Figure 3:**
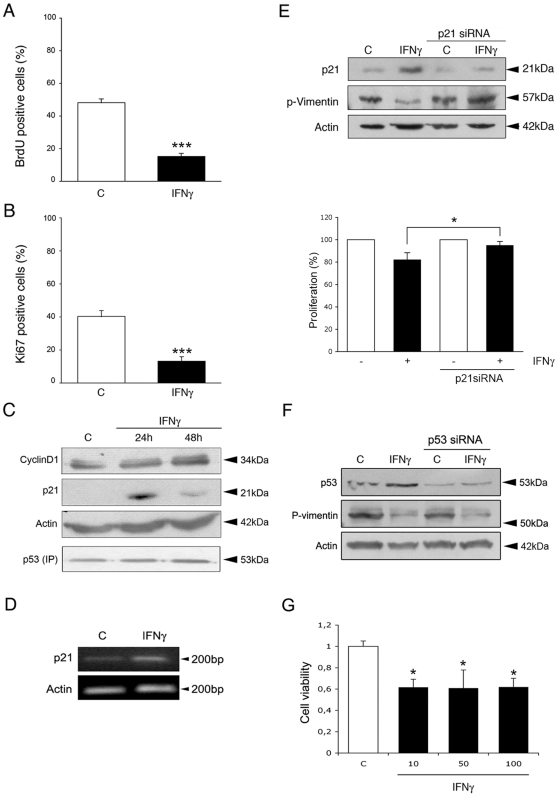
IFNγ decreases cell proliferation of NPCs via p21. NPCs were treated with 100 ng/ml IFNγ for 2 days. *p<0.05 and ***p<0.001 comparing IFNγ treated vs. controls. (**A**) 10 µM BrdU was added to the cultures to label DNA and immunostaining done using anti-BrdU antibodies. Values are means±SD, n = 4. (**B**) Immunostaining using antibody against Ki67. Values are means±SD, n = 4. (**C**) Immunoblots. Cells were treated for 24 h. Note upregulation of p21 with no change in cyclinD1. Immunoprecipitation (IP) of p53 was performed prior to blotting. (**D**) RT-PCR for p21 was done as described. Typical experiment is shown and was repeated 3 times. (**E**) NPCs were treated with siRNA against p21 for 2 days followed by IFNγ for 24 h Upper panel, Immunoblots showing p-vimentin as a measure of cell proliferation. IFNγ decreased p-vimentin in control but not in p21-siRNA treated cells. Lower panel, BrdU labeling was decreased in control but not in p21-siRNA treated cells. (**F**) NPCs were treated with siRNA against p53 followed by IFNγ as above. IFNγ reduces cell proliferation in p53-siRNA treated cells. (**G**) p53 gene deleted mouse embryonic fibroblasts were treated with 10–100 ng/ml IFNγ for 2 days and cell viability determined as described in Methods.

A decrease in cell viability may be due to reduced cell proliferation and/or enhanced cell death. Data showed that IFNγ significantly reduced the number of BrdU labeled NPCs (Fig. 3A). Similar results were obtained using antibodies against the proliferation antigen Ki67 (Fig. 3B). These results show that IFNγ decreases cell proliferation of NPCs that contributes to the reduced number of NPCs after IFNγ.

Previous studies have shown that cell cycle regulators, including p53 and cyclinD1 influence cell proliferation in NPCs [11], [15], [16]. The addition of IFNγ did not influence cyclinD1 nor p53 levels, as shown by immunoblotting (Fig. 3C). However, IFNγ increased the levels of p21 protein in the NPCs (Fig. 3C), which was also observed using RT-PCR (Fig. 3D). To study p21 more closely, we employed silencing RNA (siRNA) against p21. Treatment with p21 siRNA reduced the effect of IFNγ on cell proliferation as shown here by analyzing phospho-vimentin (p-vimentin) levels and the number of BrdU labeled cells (Fig. 3E) [17]. In contrast, lowering p53 levels by siRNA did not influence the decrease in cell proliferation caused by IFNγ (Fig. 3F). This shows that p53 is indispensable for the IFNγ-induced decrease in cell proliferation in the NPCs. In line with this, we observed that the number of p53 deficient mouse embryonic fibroblasts was also reduced by IFNγ, showing a p53 independent action for IFNγ to regulate cell viability (Fig. 3G).

### IFNγ induces cell death and activates caspase-3 in NPCs

**Figure 4:**
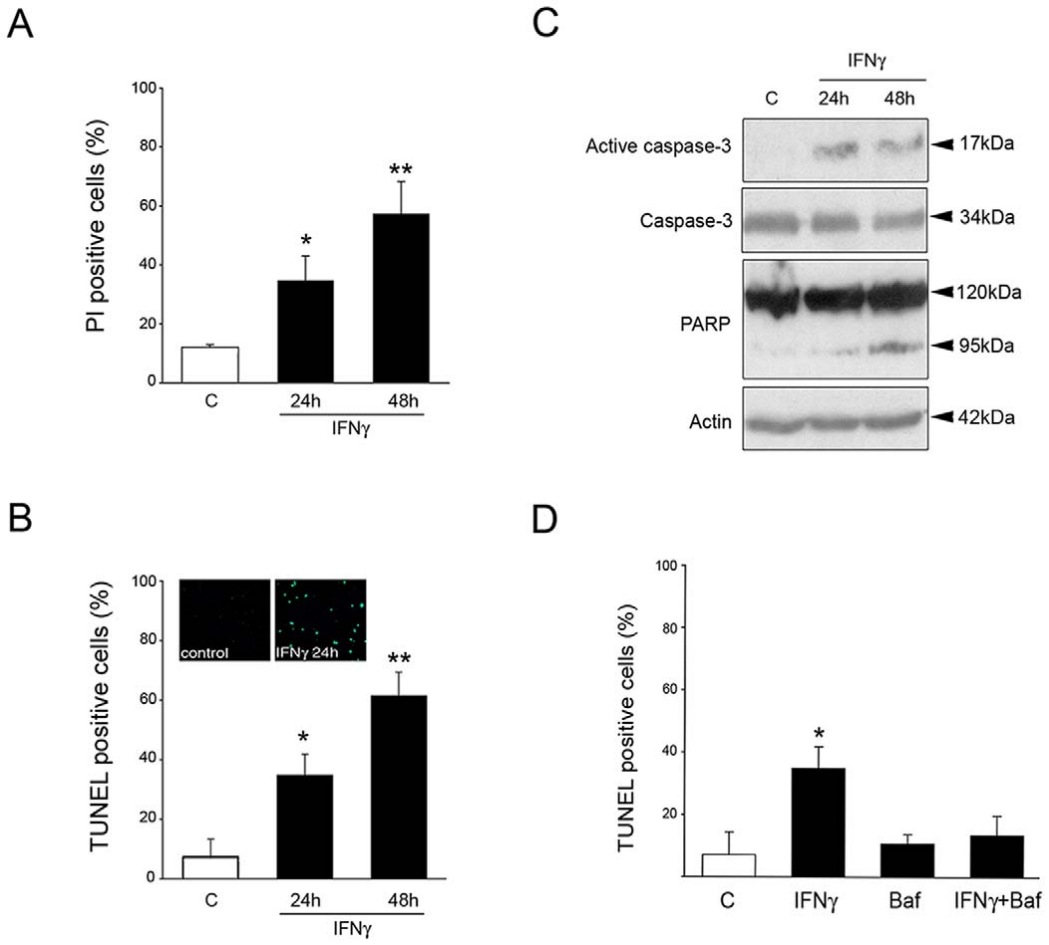
IFNγ induces cell death in NPCs via caspase-3. NPCs were treated with 100 ng/ml IFNγ for different time periods and analyzed further. *p<0.05 and **p<0.01 comparing IFNγ treated vs. controls. (**A**) Number of propidium iodine positive cells was determined by counting. Values are means±SD, n = 4. (**B**) Number of TUNEL positive cells. Values are means +SD, n = 3. Inset show typical labeling in control and IFNγ treated cells. (**C**) Immunoblot. Note increased active caspase-3 (17 kDa band) and cleaved PARP (89 kDa). Typical blot is shown and was repeated three times. (**D**) Effect of caspase inhibitor. NPCs were incubated for 24 h with 50 µM BAF in conjunction with IFNγ. Values are means +SD, n = 3.

Cell cycle analyses of NPCs revealed that IFNγ decreased the S-phase together with an increase in the sub-G0 phase (data not shown). We therefore studied whether enhanced cell death can contribute to reduced cell viability of NPCs observed with IFNγ. Staining of cells using propidium iodide or Terminal deoxynucleotidyl transferase-mediated biotinylated UTP nick end labeling (TUNEL) to analyze DNA fragmentation showed that IFNγ increased cell death of NPCs (Fig. 4. A, B). To study the mechanism involved, we analyzed caspase-3, a key caspase involved in cell death. Immunoblotting showed that IFNγ caused cleavage of caspase-3 and its downstream substrate, poly-ADP ribose polymerase (PARP) (Fig. 4C). Addition of BAF, a large spectrum caspase inhibitor, reduced cell death induced by IFNγ (Fig. 4D). Immunostaining showed that cytochrome-c was present in the cytoplasm of IFNγ-treated NPCs but not in control cells (data not shown). The release of cytochrome-c is controlled by the action of Bcl-2 family proteins on mitochondria [18]. As shown below, IFNγ increased the levels of the pro-apoptotic protein PUMA in NPCs.

### The neuropeptide PACAP increases cell viability of IFNγ-treated NPCs

**Figure 5:**
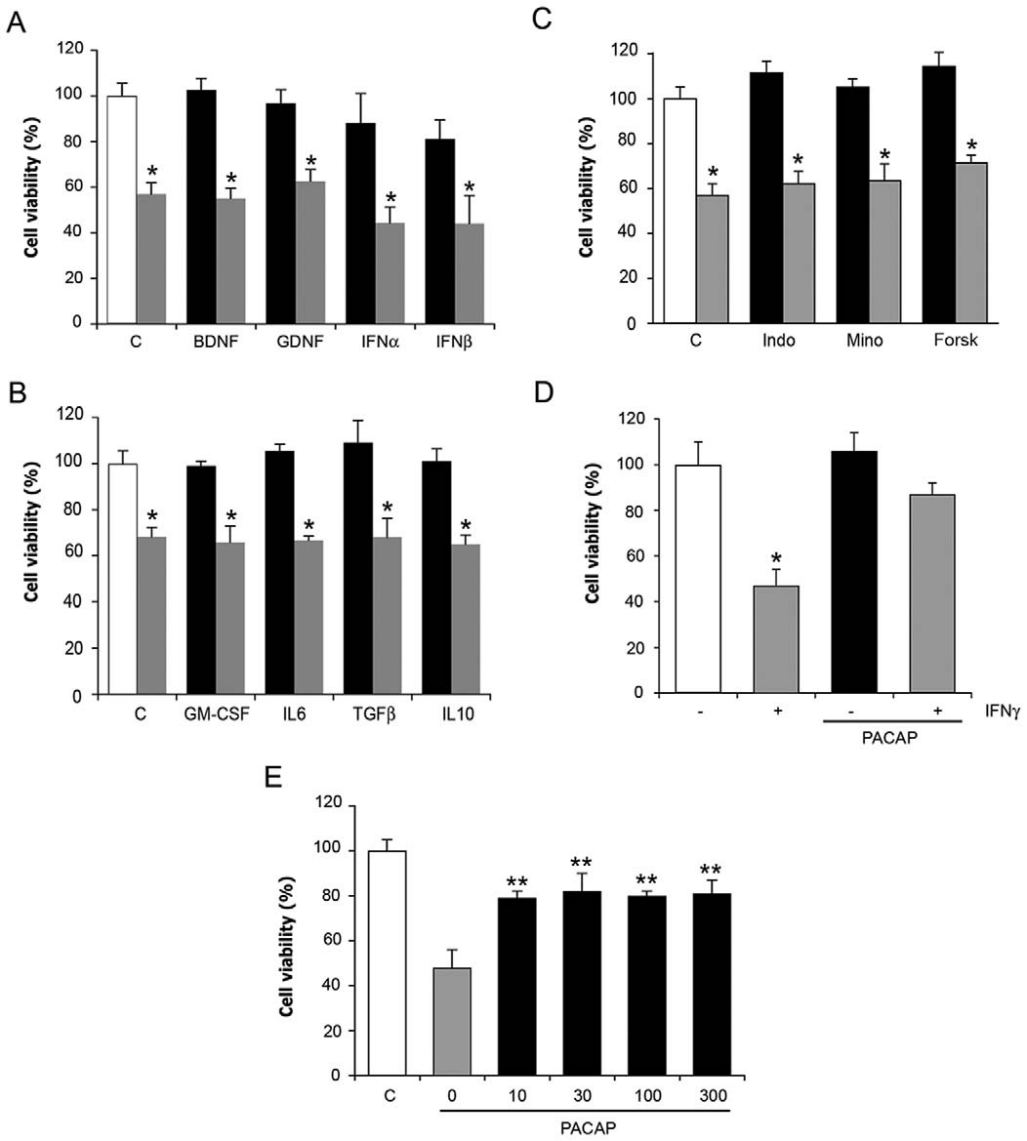
Effects of different factors in IFNγ-treated NPCs. NPCs were untreated (white bars) or treated for 24 h with various factors alone (black bars) or together with 100 ng/ml IFNγ (grey bars). Cell viability was assayed by MTT. Values are means±SD, n = 4. *p<0.05 for IFNγ treated vs. controls. (**A**) BDNF and GDNF were used at 50 ng/ml and IFNs at 100 ng/ml. Values are means±SD, n = 4. No increase in viability by the factors. (**B**) Different cytokines were used at 50 ng/ml. No increase in viability by the factors. (**C**) 100 ng/ml Indometacin (Indo), 100 ng/ml Minocyclin (Mino), and 1 µM Forskolin (Forsk) were used. No increase in viability by the compounds. (**D**) 100 ng/ml PACAP increased cell viability reduced by IFNγ. (**E**) Dose response curve for PACAP. **P<0.01 for PACAP+IFNγ vs. IFNγ.

To search for putative rescue factors to counteract the negative effects of IFNγ we incubated NPCs in the presence of various proteins and cytokines with receptors on NPCs. The results showed that none of the growth factors examined, including brain-derived neurotrophic factor (BDNF) was able to increase viability of NPCs compromised by IFNγ (Fig. 5A). In addition, IFNα or IFNβ did not counteract the effect of IFNγ (Fig. 5A). Cytokines, including IL-10 and TGFβ, which have an immune-suppressive function, had no protective effect in this system, nor did anti-inflammatory drugs including indomethacin (Fig. 5B,C). Analyses of neuropeptides that are endogenous factors in brain tissue revealed that PACAP [19] increased cell viability (Fig. 5D). PACAP efficiently counteracted the effect of IFNγ at nM concentrations that is known to activate the high affinity PACAP receptor, PAC1 (Fig. 5E).

### Mechanisms of PACAP action for cell rescue

**Figure 6:**
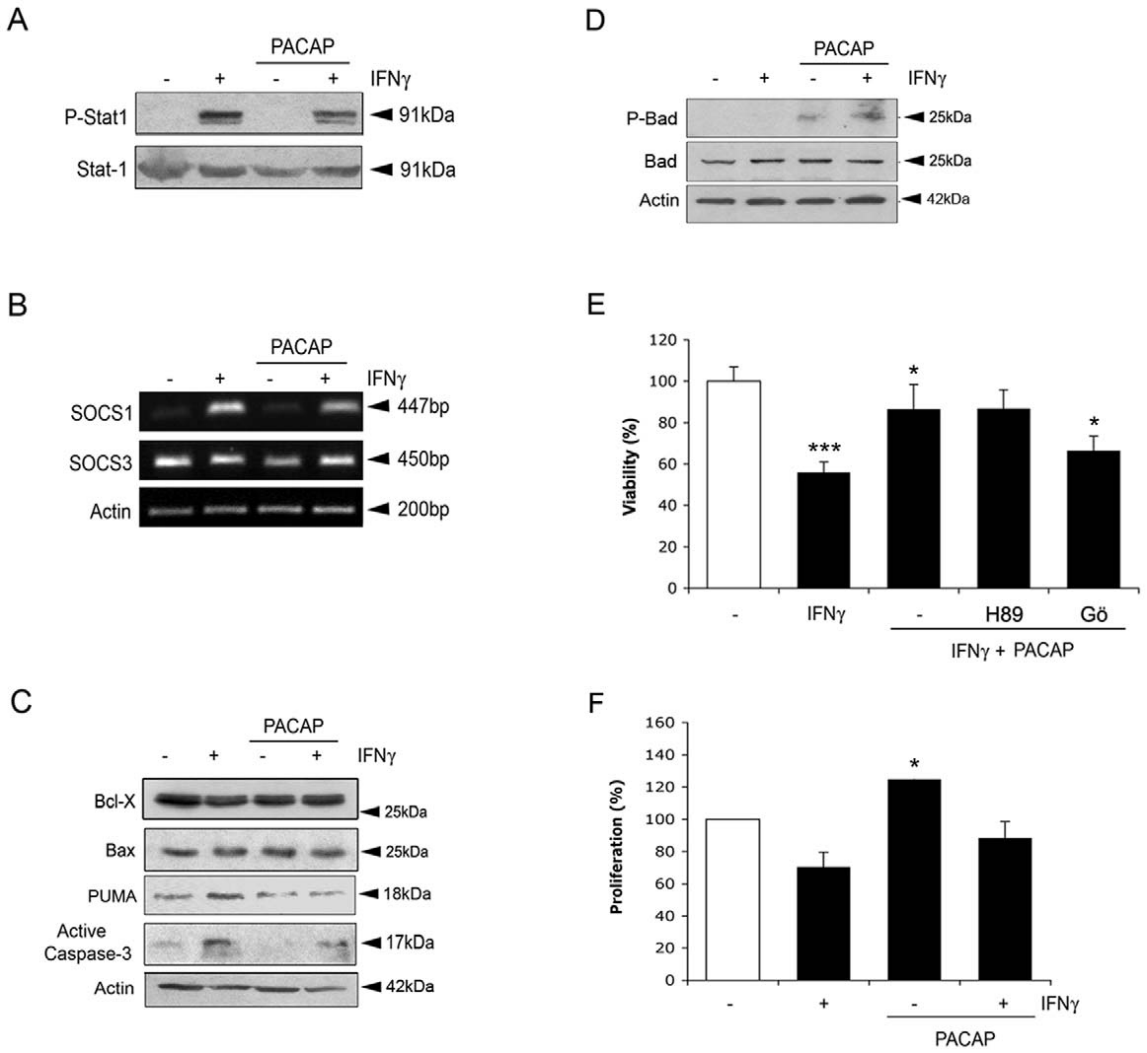
Effect of PACAP on NPCs. NPCs were treated for 24 h with 100 ng/ml IFNγ alone or in conjunction with 100 ng/ml PACAP. (**A**) Immunoblot. Cells were treated for 2 h. PACAP did not influence levels of p-Stat1 induced by IFNγ. (**B**) RT-PCR. SOCS-1 and –3 levels are not influenced by PACAP. IFNγ upregulated SOCS1. (**C**) Immunoblots. IFNγ increased whilst PACAP decreased levels of PUMA. PACAP also decreased active caspase-3 (17 kDa band). Typical blot is shown and was repeated three times. (**D**) Immunoblots. PACAP increased levels of p-Bad. Typical blot is shown and was repeated three times. (**E**) Effects of the kinase inhibitors on cell viability measured by MTT. 1 µM Gö6976 reduced the beneficial effect of PACAP whereas 10 µM H89 had no effect. Values are means ±SD, n = 4. *** p<0.005 for IFNγ vs. controls, and *p<0.0 for IFNγ+PACAP vs. IFNγ, and for GÖ+ IFNγ+PACAP vs. IFNγ+PACAP. (**F**) BrdU labeling. PACAP increases cell proliferation significantly in control but not in IFNγ treated cells. Values are means±SD, n = 4. * p<0.05 for PACAP vs control cells, n = 3.

IFNγ signaling is tightly controlled at different levels, including dephosphorylation of p-STAT1 and the induction of the suppressor of cytokine signaling (SOCS) proteins [20]. PACAP did not influence the level of IFNγ receptors (data not shown), nor the phoshorylation of STAT1 induced by IFNγ (Fig. 6A). The level of SOCS1 increased in the NPCs by IFNγ, whereas those of SOCS3 did not (Fig. 6B).

Studies of cell death regulators showed that PACAP did not influence the anti-apoptotic protein Bcl-X in the NPCs, but decreased the level of the pro-apoptotic protein PUMA that was upregulated by IFNγ (Fig. 6C). PACAP also increased the phosphorylation of Bad at serine-112 (Fig. 6D), which leads to inactivation of this pro-apoptotic protein [21].

To study the signaling pathways underlying the effect of PACAP, we employed inhibitors against protein kinase A (PKA) and protein kinase C (PKC). The PKC inhibitor, Gö6976, reduced the beneficial effect of PACAP on cell viability, whilst the PKA inhibitor, H89 did not (Fig. 6E). These results show that the effect of PACAP in increasing cell viability in NPCs involves PKC.

PACAP has previously been shown to enhance cell proliferation in adult neural stem cells [22], [23]. We observed that PACAP increased proliferation of control NPCs, but the effect was not statistically significant in NPCs treated with IFNγ (Fig. 6F). Therefore in this situation PACAP acts mainly as a survival promoting peptide reducing the inhibitory effect of IFNγ.

### Microglia affect NPCs via IFNγ

**Figure 7:**
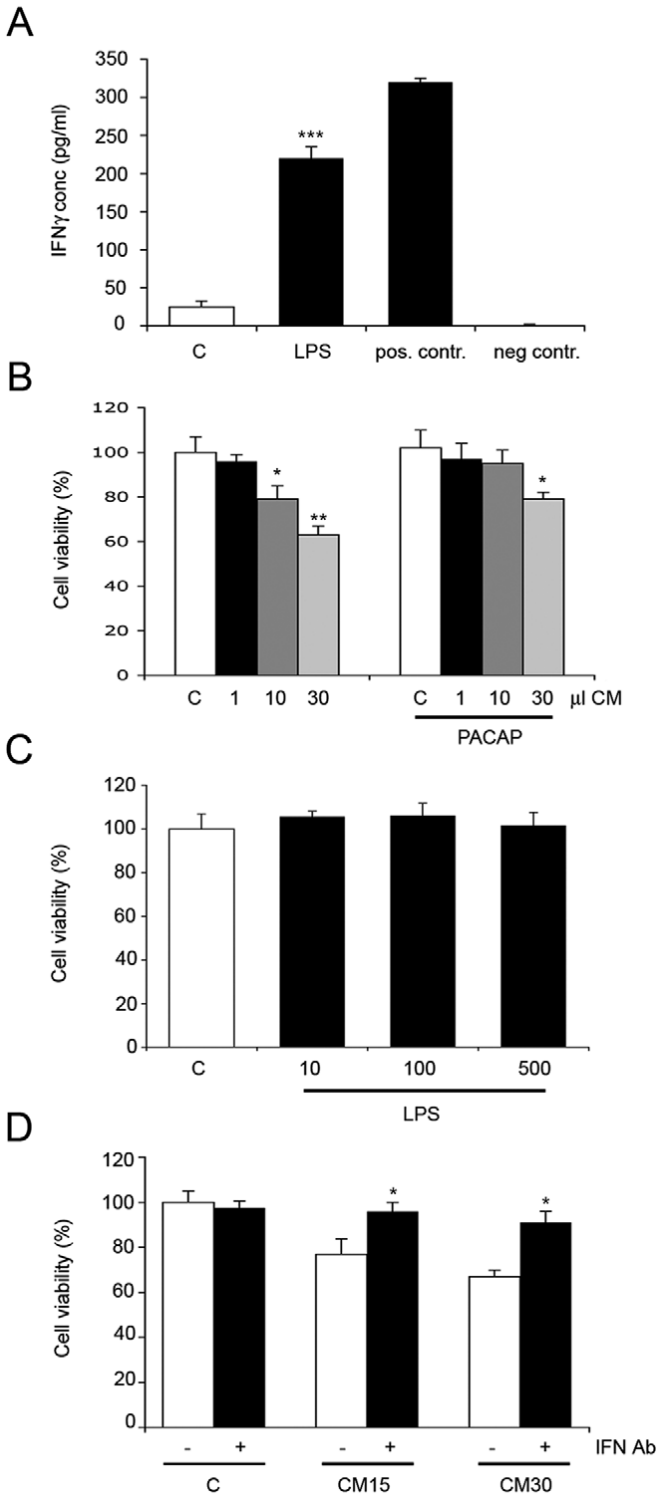
Microglia-conditioned medium decreases NPC viability. Microglial cells from rat brain were cultured and treated as described in Methods. (**A**) Activation of microglia using 500 ng/ml LPS. IFNγ was studied using ELISA as described in Methods. Positive and negative controls were from the Quantikine kit. ***p<0.001 vs. controls, n = 4. (**B**) Left panels, 30 µl medium from control microglia (C) or 1–30 µl conditioned-medium (CM) from LPS-treated microglia was added to 100 µl NPCs cultures. Values are means ±SD n = 3. *p<0.02 and **p<0.05 for 10–30 µl CM vs. C Right panels, 50 ng/ml PACAP was added to half the cultures and cell viability assayed after 24 h using MTT. *p<0.05 for 30 µl PACAP+CM vs. C. (**C**) 10–500 ng/ml LPS was added directly to NPCs for 24 and cell viability studied. No change with LPS alone. (**D**) NPCs were incubated with 30 µl control medium (C) or with 15 (CM15) or 30 µl (CM30) medium from LPS-treated microglia in absence or presence of 0.25 µg/ml blocking anti-IFNγ antibodies (IFN-Ab). Values are means ±SD n = 3. *p<0.02 for IFN-Ab vs. CM-treated cultures.

In the brain, microglial cells represent a defense system that takes part in inflammation and in immune reactions. Activation of microglial cells is followed by increased production of various cytokines. We observed that activation of rat microglial cells by lipopolysaccaride (LPS) led to increased levels of IFNγ in the culture medium as shown by ELISA (Fig. 7A). Addition of the LPS-conditioned medium to NPCs reduced cell viability by up to 40% compared with media from control cells (Fig. 7B left panels). Supplementation of the conditioned media with 50 nM PACAP counteracted the decrease in viability of NPCs (Fig. 7B right panels). LPS itself had no effect on NPCs viability excluding nonspecific effects of the compound (Fig. 7C). Incubation of the microglia-derived medium with anti-IFNγ blocking antibodies largely reduced its negative effect on cell viability (Fig. 7D). These results show that microglia produce IFNγ and that PACAP is able to counteract the decrease in cell viability observed with the microglia-conditioned medium.

## Discussion

The present results show that IFNγ influences NPCs in two ways by decreasing cell proliferation and by increasing cell death. IFNγ affected cell proliferation by inducing expression of p21, and cell death by influencing various Bcl-2 family proteins and caspase-3 activation. Previous studies have shown that cell cycle regulators including p53 and cyclinD1 are important for cell proliferation of NPCs [11], [15], [16], but we observed no significant change in these proteins in IFNγ-treated NPCs. In addition, IFNγ reduced cell proliferation also in p53 deficient fibroblasts and in NPCs after downregulation of p53 by siRNA. p21 is a downstream target for p53 but the protein may be regulated also by other factors. We observed that IFNγ increased the expression of p21 in NPCs, leading to a decrease in cell proliferation. In line with this, IFNγ has been shown to regulate p21 in human breast cancer cells via transcriptional upregulation [24].

IFNs are divided into two major classes: type-1 IFNs, with the structurally related IFNα, IFNβ, and type-2 IFN, with IFNγ [12]. The IFNs bind to specific IFN receptors on target cells and activate gene transcription through the STAT and Janus tyrosine kinase signaling pathway [25]. IFN receptors are predominantly expressed by the immune cells, but in the brain astrocytes and neurons also show expression [12], [14]. Administration of IFNs including IFNγ has been shown to modulate neuronal activity and to alter behavior in experimental animals [12]. The exact roles of IFNγ in brain physiology and its cellular targets are, however, not fully understood.

In the present study, we observed a high abundance of IFNγ receptors in neural precursor cells in neuroepithelium during rat embryonic development and in NPCs cells in culture. IFNγ activated STAT-1 signaling and the nuclear translocation of the protein. STAT-1 in turn activates transcription of downstream genes including those affecting inflammation, cell signaling and cell survival [20], [25]. IFNγ was found to increase levels of the BH-3 only protein PUMA in the NPCs, which can induce the mitochondrial pathway of cell death [18], [21], [26]. In IFNγ treated cells cytochrome-c was released into the cytoplasm with activation of caspase-3, the cleavage of PARP and with increased DNA fragmentation. Incubation with the caspase-inhibitor, BAF protected NPCs against cell death induced by IFNγ. These results show that IFNγ influences cell death in NPCs by influencing a subset of death-regulating proteins including PUMA with the activation of caspase-3.

The observed decrease in cell viability induced by IFNγ may negatively influence the survival and recruitment of NPCs after brain injury or in different diseases. We therefore studied a variety of growth factors and cytokines with described receptors on NPCs for their ability to counteract the effects of IFNγ. Of the many factors examined, the neuropeptide PACAP had the largest effect in increasing cell viability. PACAP is a member of the vasoactive intestinal peptide/secretin/glucagon peptide family [19] and is present in embryonic and adult brain tissue [27]–[32]. PACAP binds to its high-affinity PAC1 receptor that is expressed by different target cells [33], [34], including NPCs [27]. Previous studies have shown that PACAP increases proliferation/survival of adult neural stem cells [2], [23], [35]. PACAP also increases neuronal survival in different systems [31], [36], however, this action may involve other growth factors, such as insulin growth factor-1 [33] and interleukin-6 (IL-6) [37]. We observed that neither IL-6 nor the other neurotrophic factors studied counteract the IFNγ-induced loss of cell viability. In contrast, PACAP at low concentrations exerted a robust protection against cell degeneration of NPCs induced by IFNγ, The enhanced cell viability induced by PACAP was accompanied by increases in p-Bad that can inhibit the mitochondrial mediated cell death [21]. In line with this, the activation of caspase-3 by IFNγ was reduced in PACAP-treated NPCs. This data suggests that alterations in expression and phosphorylation of Bcl-2 family proteins may underlie the increase in NPC viability induced by PACAP. Inhibitor studies further showed that increased cell viability by PACAP was mainly mediated by an activation of PKC in NPCs. Molecular cloning has revealed the existence of six different splice variants of the PAC1 receptors, which can activate cell signaling of PACAP either through PKC or PKA [33]. These splice variants are also present in neural stem cells and mediate various cellular responses to PACAP [22], [23], [35].

Studying the possible crosstalk between PACAP and IFNγ, we observed that PACAP did not influence STAT1 signaling nor did PACAP affect the IFNγ receptor levels in NPCs (Fig. 6). IFNγ receptor signaling is a complex process controlled by several factors including the SOCS proteins [20]. As studied here, SOCS-1 and -3 are expressed by NPCs but these proteins were not altered by PACAP. Although the signaling cascades induced by IFNγ and PACAP do not directly overlap, some genes including the Bcl-2 family proteins are differentially regulated by these two factors. Future studies using gene profiling will reveal which other proteins are regulated by PACAP and by IFNγ in the NPCs.

Apart from cell survival, PACAP may increase viability of IFNγ-treated NPCs by enhancing cell division [22]. We observed a slight increase in cell proliferation by PACAP after IFNγ treatment but this was not statistically significant as shown using BrdU labeling (Fig. 6G). We therefore conclude that the major effect of PACAP on IFNγ-treated NPCs was to promote cell survival by inhibition of caspase-3 activation and increasing p-Bad levels.

Brain tissue is usually though as being protected from immunological reactions in the body by the presence of the blood brain barrier. The immune and nervous systems interact with each other in conditions characterized by increased leakage of the blood brain barrier that occurs after an immune challenge or in brain inflammation. The activation of immune cells occurs in various human brain disorders and neurodegenerative diseases [1]. The levels of IFNγ increase in brain in different diseases, such as multiple sclerosis and encephalitis and following immune reactions. IFNγ may be produced by invading immune cells or by resident microglial cells that are activated during the inflammatory process. Microglia have recently been shown to interact with neural stem cells and these cells may mediate both positive [38], [39] and negative effects on NPCs (this study). NPCs in turn exert potent anti-inflammatory actions *in vivo* [40], [41]. The final outcome of interaction between NPCs and inflammatory cells probably depends on the amount of secreted cytokines at each moment. We observed that LPS-activated microglia cells produce IFNγ into the culture medium and this negatively affected the NPCs. Experiments using blocking antibodies against IFNγ showed that a part of the activity in the medium is due to IFNγ, although other factors cannot be excluded. In line with our data, it was recently reported that the addition of IFNγ inhibited neurosphere formation in adult murine NPCs [42]. Previous studies have shown that PACAP may have potent anti-inflammatory function in regulating the production of pro-inflammatory mediators [43]. This has recently been confirmed in PACAP gene deficient mice that showed an increased expression of pro-inflammatory cytokines including IFNγ [44]. We show here that PACAP directly acts on NPCs to counteract the effects of IFNγ. PACAP is able to pass the blood-brain barrier [45]. PACAP may therefore be useful in treatment of brain inflammation and to enhance recruitment of endogenous NPCs after injury and conditions with high IFNγ production. Due to its robust cell survival effects on NPCs PACAP may also be considered as an adjuvant treatment in different NPC transplantation studies.

## Materials and Methods

### Animals

Wistar rats were obtained from Harlan (Horst, The Netherlands). All experiments were approved by the local ethical committee and performed in accordance with the European Communities Council Directive (86/609/EEC).

### Cell culture

NPCs were prepared from embryonic (E) 17-old rat brains as described [11], [46], [47]. Cells were cultured in medium containing 20 ng/ml epidermal growth factor (EGF) (PeproTech, Rocky Hill, NJ), and B27 supplement in DMEM/F-12 (Gibco, Invitrogen Carlsbad, CA, USA). Neurospheres were grown for 5 days, gently dissociated, and collected by centrifugation for 5 min at 1500 rpm. The cells were resuspended into appropriate volume of medium containing EGF, different IFNs (PeproTech), PACAP-38 (Bachem, Bubendorf, Switzerland) and growth factors and cytokines added as indicated. These included BDNF (PeproTech) G-CSF; GM-CSF, IL-6, IL-10, CNTF (Sigma, St. Louis, MO, USA) that have shown to act on NPCs. Broad range caspase inhibitor boc-aspartyl (Ome)-fluoromethylketone (BAF) was from Calbiochem (San Diego, CA, USA) and the compounds, indomethacin, minocyclin and forskolin and kinase inhibitors, H89 and Gö6976 were all from Sigma.

Microglial cells were prepared from newborn rat brain essentially as described [13]. and kept in culture for up to three weeks in Dulbecco's modified Eagle's medium (DMEM)/F12 medium (Gibco) supplemented with 10% fetal bovine serum, and penicillin/streptomycin at 37°C in 5% CO_2_ atmosphere. Cells were stimulated for 24 h using bacterial lipopolysaccharides (LPS, Sigma) and conditioned medium (CM) collected and the amount of IFNγ analyzed using ELISA and the rat Quantikine assay (R&D Systems, Minneapolis, USA). Blocking anti-IFNγ antibodies was from R&D Systems. Unstimulated CM was used as control. CM from microglial cells was added to cultures of NPCs and cell viability determined as below. p53 gene deleted mouse embryonic fibroblasts (kind gift of M Laiho) were cultured in DMEM/10% fetal bovine serum and stimulated with 100 ng/ml mouse IFNγ (PeproTech).

### NSC viability, cell death and proliferation assays

NSCs were cultured in 96-well cell culture dishes (70,000 cells per well; Costar 3599; Corning) in the presence of 20 ng/ml EGF and different concentrations of IFNγ and PACAP. To estimate the viability of cells, we used the MTT [3-(4,5-dimethylthiazol-2-yl)-2,5-diphenyltetrazolium bromide (Sigma) assay as described previously [11], [46], [47]. For TUNEL staining of DNA breaks the In Situ Cell Death Detection Kit (Roche, Basel. Switzerland) was used as described [11], [48], [49]. BrdU (Sigma) labeling and immunostaining using the Ki67 antibody (1∶300; BD Biosciences, Franklin Lakes, NJ, USA) were performed to estimate the number of proliferating cells [11], [49]. Cell cycle analyses were done using flow cytometry using a FACS calibur flow cytometer [50]. To estimate the capacity for self-renewal an equal number of NPCs (5000 cells) were incubated for 3 days in the absence and presence of 100 ng/ml IFNγ and the number of neurospheres counted [11], [47].

### Immunochemistry

NPCs plated in 24-well culture dishes coated with poly-DL-ornithine (50 µg/ml), fixed for 20 min using 4% paraformaldehyde, and blocked for 1 h using 3% BSA in PBS/0.1% Triton X-100. The following primary antibodies were added overnight at 4°C: IFNγR2 (diluted 1∶1500; Abcam, Cambridge, UK), cytochrome-c (1∶200 BD Bioscience), phospho-STAT1 (1∶1000; Cell Signaling Technology, Inc., Danvers, MA, USA), and nestin (1∶1000; R&D Systems). Secondary Alexa 488 and Alexa 594 flurosecent antibodies (1∶500; Invitrogen) were added for 1 h in PBS in 1% BSA and 0.1% Triton X-100. The number of immunoreactive cells in each well was counted using fluorescent microscopy in four independent fields.

For staining of neuroepithelium, 15 µm sections from E17 old rats were cut using a Leitz (Wetzlar, Germany) microtome and placed on SuperFrost Plus glass slides [11]. Anti-IFNγR2 (diluted 1∶500) or anti-nestin (1∶100) antibodies were added overnight together with followed by washing with PBS. Appropriate secondary antibodies were added for 1 h and sections were washed and mounted with gel mounting medium (Gel Mount; Sigma).

### Immunoblots

Cells were lysed in a buffer containing 50 mM Tris-HCl (Ph 7.4), 1% NP-40, 0.25% natriumdeoxycholate, 150 mM NaCl, 1 mM EDTA, and protease inhibitors (Roche) [11], [48]. In some experiments phosStop solution (Roche) was added to inhibit phosphatases. 40–80 µg total protein were separated using SDS–PAGE, transferred to nitrocellulose membranes (Hybond-C Extra, Amersham), blocked for 1 h in 5% skim milk, and incubated overnight at 4°C with primary antibody in blocking buffer. Antibodies used were: cyclin D1 (1∶750) and Bax (1∶250) from Santa-Cruz (CA, USA), STAT1 (1∶400), p-STAT1 (1∶500); caspase-3 (1∶1000), cleaved caspase-3 (1∶1000); PARP (1∶2000), p53 (1∶1000), Bad (1∶1000), p-Bad (1∶500; phospho-Ser112), and Puma (1∶1000) all from Cell Signaling, Bcl2 (1∶400) and Bcl-xL (1; 1000) from BD Bioscience, IFNγR-2 (1∶3000; Abcam), p21 (1∶200; Millipore, MA, USA), p-vimentin (1∶2000; phospho-Ser55 Assay Designs, Ann Arbor, MI, USA), and β-actin (1∶2000, Sigma). Appropriate peroxidase-conjugated antibodies (1∶2500, Jackson Immunoresearch, Newmarket, Suffolk, UK) were added for 1 h and detection was performed using SuperSignal West Pico Substrate (Pierce). Quantification was performed using GelDoc (BioRad).

### Silencing RNA

100 nM siRNA construct against p53 and against p21 (Dharmacon, Lafayette, CO, USA) was tranfected using the Amaxa Nucleofector system and 5×10^6^ NPCs [48], [49]. Equal number of control and treated cells were incubated for 48 h followed by 100 ng/ml IFNγ for additional 24 h. Efficacy of downregulation was analyzed by immunblotting and the levels of p-vimentin that increases during M-phase of the cell cycle [17].

### RT-PCR

Total RNA was extracted from NPCs, cDNA and polymerase chain reaction (PCR) performed as described before [11], [47] using 30cycles of amplification and with following steps: 95°C for 30 s, 60°C for 30 s and 72°C for 60 s. Primers were: IFNγR1, forward (Fw), 5′-CGC CTG TAT CCC CTT TCT CCA T-3′ and Reverse (Rev), 5′-CAT CTT TGT TTC CGA GTC GTT GTT T-3′; IFNγR2, Fw, 5′-CGG CCG CTT GAA GGT TTT CCC ATA C-3′ and Rev, 5′-GAG GCA TCC GCT GTT GTT TCG TGA C-3′;

PAC1 receptor: Fw, 5′- GCT CTA TTT TGA TGA TGC AG-3′ Rev, 5′-CTT GCT CAG GAT GGA CAG CT-3′; p21 Fw, 5′- AGG CAG ACC AGC CTA ACA GA -3′; Rev: 5′- CAG CAC TAA GGA GCC TAC CG -3′; β-actin, Fw, 5′-CAC ACT GTG CCC ATC TAT GA-3′ and Rev, 5′-CCA TCT CTT GCT CGA AGT CT-3′

### Statistics

Statistical comparisons were performed using Student's t-test when comparing two groups, or one-way ANOVA followed by a Bonferroni post hoc test when comparing three or more groups.
